# Exosomes in Hypertrophic Scars and Keloids: Mechanisms and Therapeutic Potentials—A Narrative Review

**DOI:** 10.1111/jocd.70705

**Published:** 2026-01-30

**Authors:** Mengke Wu, Jianfeng Zhang, Na Xiong, Yixiao Ma, Lingling Yong, Huaxu Liu, Qin Guo

**Affiliations:** ^1^ Affiliated Hospital of Nanjing University of Chinese Medicine, Jiangsu Province Hospital of Chinese Medicine Nanjing China; ^2^ Nanjing University of Chinese Medicine Nanjing China; ^3^ Hospital for Skin Diseases, Shandong First Medical University Jinan Shandong China

**Keywords:** cell‐free therapy, exosome, fibrosis, hypertrophic scar, keloid, TGF‐β/Smad, wound healing

## Abstract

**Background:**

Hypertrophic scars and keloids, types of pathological scars, arise from dysregulated wound healing, marked by abnormal fibroblast activation and excessive extracellular matrix (ECM) deposition. Current treatments have high recurrence rates and side effects, necessitating targeted therapies. Exosomes, extracellular vesicles mediating intercellular communication, offer multi‐target regulatory potential to address scar formation complexities.

**Methods:**

This narrative review synthesizes in vitro and in vivo studies (2020–2025) from PubMed and Scopus on exosomes' role in regulating hypertrophic scars and keloids, proposing innovative therapeutic approaches.

**Results:**

Therapeutic exosomes attenuate inflammation, promote wound healing, inhibit fibrosis, and modulate the scar microenvironment. They suppress fibroblast‐to‐myofibroblast transformation, regulate collagen synthesis, and inhibit fibrotic pathways, particularly via the Transforming Growth Factor‐beta/Sma‐ and Mad‐related protein (TGF‐β/Smad) signaling pathway.

**Conclusion:**

Exosomes are a promising cell‐free therapy for pathological scars due to their multi‐target regulatory capabilities. Future research should optimize large‐scale production, standardize protocols, and develop targeted delivery systems to enable clinical translation, with validation through clinical trials.

## 1 Introduction

### 1.1 Wound Healing and Pathological Scarring

Wound healing is a coordinated process involving four overlapping phases: hemostasis, inflammation, proliferation, and remodeling. Dysregulation of this sequence can lead to pathological scars, such as hypertrophic scars and keloids [1]. Unlike physiological repair, pathological scarring is characterized by a prolonged inflammatory phase and a failure to regulate subsequent proliferation. The hallmark features include aberrant fibroblast activation and excessive ECM deposition [2].

#### 1.1.1 Hemostasis

Triggered immediately upon injury, this phase involves platelet activation and thrombus formation to seal the wound. Platelets also release growth factors, such as TGF‐β, which initiate tissue repair [3]. In pathological scarring, an exaggerated hemostatic response leads to an overabundance of pro‐inflammatory and pro‐fibrotic signals, setting a dysfunctional foundation for the subsequent healing stages [3].

#### 1.1.2 Inflammation

Typically resolving within three days, the inflammatory response recruits neutrophils and macrophages to clear pathogens and necrotic debris [4]. In hypertrophic scars, this phase is significantly prolonged. Persistently activated macrophages continue to release pro‐fibrotic factors, preventing inflammatory resolution and overdriving the proliferative process [4].

#### 1.1.3 Proliferation

Beginning several days post‐injury, this phase focuses on angiogenesis, collagen deposition, granulation tissue formation, and re‐epithelialization [5]. Driven by persistent inflammation, fibroblasts become hyperactivated and differentiate into myofibroblasts, leading to excessive ECM accumulation [5]. While re‐epithelialization aims for wound closure, any impairment in this process leaves the dermis exposed, further exacerbating fibrosis and scar formation [6].

#### 1.1.4 Remodeling

In the final phase, normal repair achieves a balance between ECM synthesis and degradation. In pathological scars, however, persistent myofibroblast activity causes collagen synthesis to far outpace degradation [7]. This results in a disorganized accumulation of collagen fibers, manifesting clinically as raised, erythematous scars that are often pruritic or painful [7].

### 1.2 Clinical Burden and Therapeutic Challenges

Pathological scars cause not only aesthetic concerns but also contractures, itching, and functional impairments, significantly reducing patients' quality of life and imposing a socioeconomic burden [8].

Current treatments, such as corticosteroid injections, pressure therapy, laser therapy, and surgical excision, only partially alleviate symptoms and are limited by high recurrence rates, poor targeting, and significant side effects [9, 10, 11]. These therapies struggle to precisely target key fibrotic pathways, such as the TGF‐β/Smad signaling pathway, highlighting the need for novel therapeutic approaches [12].

### 1.3 The Introduction of Exosomes

**FIGURE 1:**
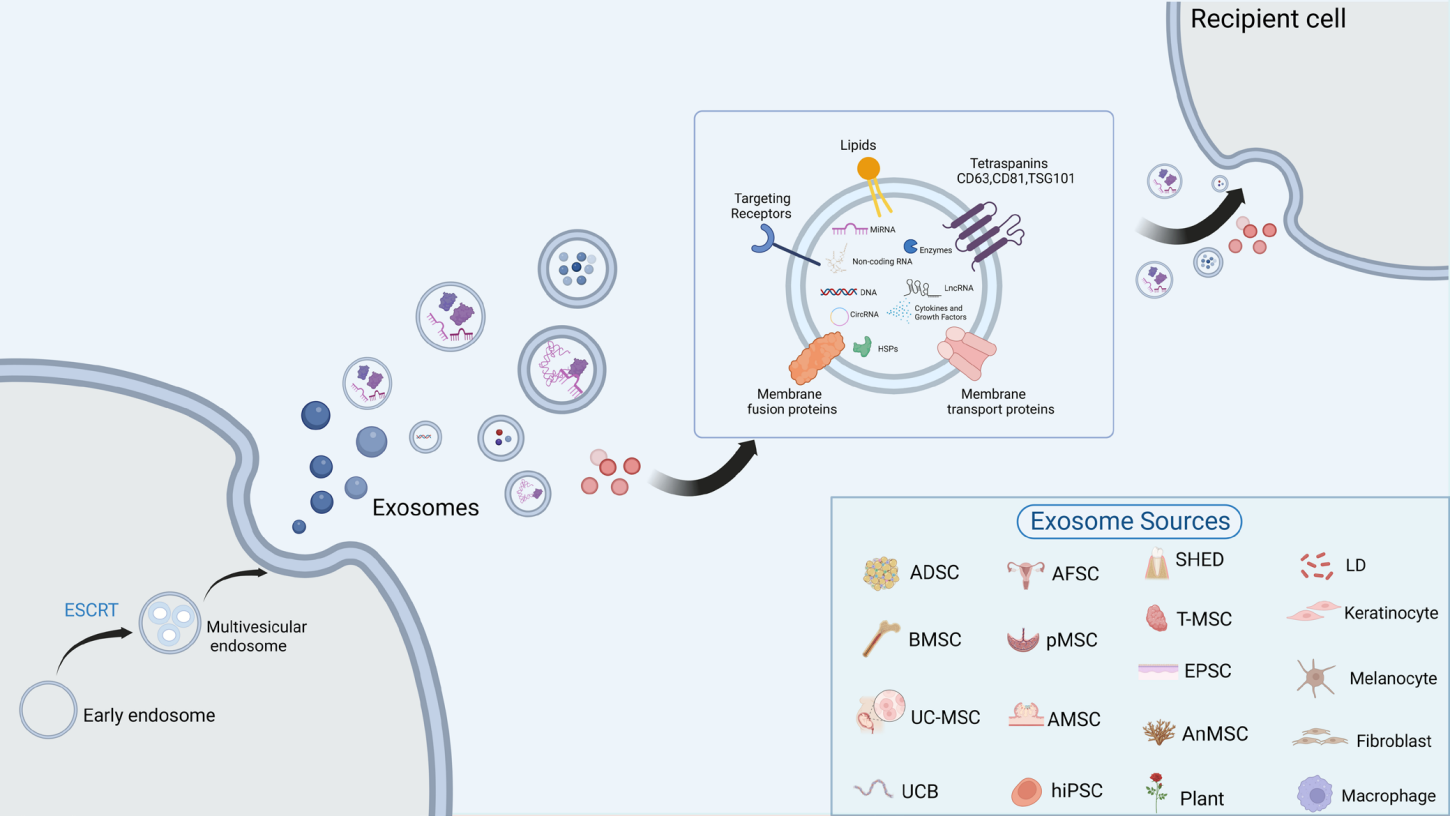
Exosome biogenesis, secretion, and delivery in pathological scar formation. ADSC, adipose‐derived mesenchymal stem cell; AFSC, amniotic fluid stem cells; AMSC, amniotic membrane stem cell; AnMSC, Antler mesenchymal stem cell; BMSC, bone marrow mesenchymal stem cell; EPSC, epidermal stem cell; hiPSC, human induced pluripotent stem cell; LD, *Lactobacillus delbrueckii* ; pMSC, placental mesenchymal stem cell; SHED, stem cells from human exfoliated deciduous teeth; T‐MSC, tonsil‐derived mesenchymal stem cell; UCB, umbilical cord blood; UC‐MSC, umbilical cord mesenchymal stem cell.

Exosomes are extracellular vesicles (EVs), 50–150 nm in diameter, formed through biogenesis involving cell membrane invagination to create early endosomes, followed by endosomal sorting complex required for transport (ESCRT)‐mediated inward budding to form multivesicular endosomes (MVEs) containing intraluminal vesicles (ILVs) [13]. Exosomes carry proteins, messenger RNAs (mRNAs), microRNAs (miRNAs), and other molecules from their source cells, serving as effective carriers for intercellular signaling [14]. Their composition varies based on cell type, functional state, and microenvironment. Exosomes deliver cargo to target cells via receptor‐mediated endocytosis (e.g., CD63, CD81, TSG101) or direct release of active molecules, regulating gene expression, signal transduction, and metabolic processes [15] (Figure 1).

Research on exosomes has surged in recent years due to their role as key mediators of intercellular communication [16]. Studies increasingly focus on exosome‐based therapies for scarring with evidence highlighting their regulatory potential in fibrotic diseases, including hepatic, renal, and pulmonary fibrosis [17, 18, 19]. These findings suggest exosomes could address the fibrotic processes underlying pathological scarring, offering a promising avenue for therapeutic development [20].

## 2 The Effect of Exosomes on Pathological Scars

Exosomes promote wound repair by delivering bioactive molecules that regulate inflammation, angiogenesis, and re‐epithelialization [21]. These properties underpin their potential in wound healing and scar therapy [22]. Exosome function depends on their source cells and microenvironment. Under physiological conditions, exosomes facilitate intercellular communication, immune regulation, and tissue regeneration through specific molecular signals [23]. In pathological conditions, such as hypertrophic scars and keloids, their cargo can activate pro‐fibrotic pathways or disrupt epidermal‐dermal interactions, leading to ECM deposition and scar formation [24]. Exosomes exhibit functional heterogeneity based on their origin, as summarized in Table 1.

**TABLE 1:** Roles of exosomes in pathological scarring.

Exosome source	Key molecules	Impact on scars	References
ADSCs	miR‐125b‐5p, miR‐194, miR‐29a	Therapeutic	[25, 26]
UC‐MSCs	NGFs	Therapeutic	[27]
SHED	miR‐1246	Therapeutic	[28]
LD	—	Therapeutic	[29]
*Rosa damascena*	miR‐1246, miR‐574‐5p	Therapeutic	[30]
Hypertrophic scar fibroblasts	miR‐21	Pathogenic	[31]
Keloid patient plasma	miR‐193a‐5p	Pathogenic	[32]
Melanocytes	miR‐7704	Pathogenic	[33]
M2 macrophages	CXCL2	Pathogenic	[34]
M2 macrophages	miR‐34a‐5p	Pathogenic	[35]

Abbreviations: ADSCs, adipose‐derived MSCs; CXCL, C‐X‐C motif chemokine ligand; LD, *Lactobacillus* *delbrueckii* ; NGFs, nerve growth factors; SHED, stem cells from human exfoliated deciduous teeth; UC‐MSCs, umbilical cord MSCs.

### 2.1 Stem Cell‐Derived Exosomes

Research on stem cell‐derived exosomes for scar formation focuses primarily on mesenchymal stem cell (MSC) sources, particularly adipose‐derived MSC exosomes (ADSC‐Exos) [36]. ADSC‐Exos reduce excessive collagen deposition by modulating the transforming TGF‐β/Smad and Notch‐1 pathways while exerting an anti‐inflammatory effect [25, 26, 37]. Umbilical cord‐derived MSC exosomes (UC‐MSC‐Exos) secrete nerve growth factors (NGFs) to enhance neural regeneration and function in scarred tissue and reduce melanin deposition by inhibiting tyrosinase activity, improving scar structure and pigmentation [27, 38]. Exosomes from stem cells of human exfoliated deciduous teeth (SHED‐Exos) activate macrophage autophagy and regulate the AKT, extracellular signal‐regulated kinase (ERK)1/2, and Signal Transducer and Activator of Transcription 3 (STAT3) pathways, promoting anti‐inflammatory and wound‐healing effects [28].

### 2.2 Bacterial Extracellular Vesicles

Bacterial EVs are an emerging area of research in scar therapy due to their unique properties. *Lactobacillus delbrueckii* EVs (LDEVs), formed by membrane budding in Gram‐positive bacteria, differ from eukaryotic exosomes (released via multivesicular body exocytosis) but share a similar 80–130 nm lipid bilayer structure and molecular delivery capabilities [29]. Compared to MSC‐derived exosomes (MSCs‐Exos), LDEVs offer advantages: (1) ease of culture and rapid proliferation, enabling large‐scale production; (2) avoidance of ethical concerns associated with stem cells; and (3) lack of human leukocyte antigen expression, reducing immune rejection risk. LDEVs inhibit uncontrolled fibroblast proliferation by modulating the mitogen‐activated protein kinase (MAPK) signaling pathway, promoting wound healing [29].

### 2.3 Plant‐Derived Exosomes

Plant‐derived exosomes represent a novel therapeutic frontier in cutaneous repair and scar management [39]. Specifically, exosomes derived from *Rosa damascena* stem cells (RSCEs) are enriched with bioactive molecules, including the Let‐7 family, miR‐1246, and miR‐574‐5p [30]. These RSCEs appear to exert a biphasic effect on wound healing: in the early stages, they accelerate tissue repair and re‐epithelialization by promoting fibroblast proliferation and migration; during later stages, they modulate ECM remodeling to balance collagen synthesis and degradation, thereby mitigating excessive fibrosis [30].

In vitro evidence further supports the anti‐inflammatory potential of RSCEs, specifically through the suppression of the pro‐inflammatory cytokine IL‐6 [30]. These mechanistic findings are mirrored in clinical settings, where case reports indicate that combining RSCEs with microneedling therapy significantly enhances the flattening and textural improvement of traumatic scars [40, 41].

Compared to animal‐ or human‐derived stem cell therapies, Plant‐derived exosomes offer distinct advantages in biocompatibility and safety. Their natural origin minimizes immunogenic risks and bypasses the ethical complexities often associated with traditional cell‐based treatments, making them a highly attractive alternative for regenerative medicine [39].

### 2.4 Fibroblast‐Derived Exosomes

Exosomes from pathological scar fibroblasts contribute to scar formation by carrying pro‐fibrotic factors, such as miR‐21, miR‐193a‐5p, and TGF‐β1. These molecules activate the TGF‐β/Smad signaling pathway, inhibit keratinocyte migration, and impair epidermal barrier function, promoting scar development [24, 31, 32]. Additionally, these exosomes suppress melanin synthesis in epidermal melanocytes, revealing a cross‐cellular mechanism underlying scar pigmentation abnormalities [42].

### 2.5 Melanocyte‐Derived Exosomes

Melanocytes, responsible for skin pigmentation, regulate scar formation via paracrine mechanisms. Melanocyte‐derived exosomes, enriched with bioactive molecules like miR‐7704, activate the TGF‐β/Smad signaling pathway, promoting fibroblast proliferation and collagen synthesis, which leads to excessive ECM deposition [33].

### 2.6 Macrophage‐Derived Exosomes

M2 macrophage‐derived exosomes promote fibrosis by activating fibroblast autophagy through the C‐X‐C motif chemokine receptor (CXCR)2/CXCR7/mammalian target of rapamycin (mTOR) signaling pathway [34]. In a urethral scar model, these exosomes target Silent Information Regulator Two Homolog 1 (SIRT1), accelerating fibrosis, indicating tissue‐specific effects [35]. The role of macrophage‐derived exosomes depends on the cytokine network, target cell characteristics, and pathological stage.

## 3 Regulatory Mechanisms of Exosomes in Pathological Scar Formation

### 3.1 Regulation of Inflammation

**FIGURE 2:**
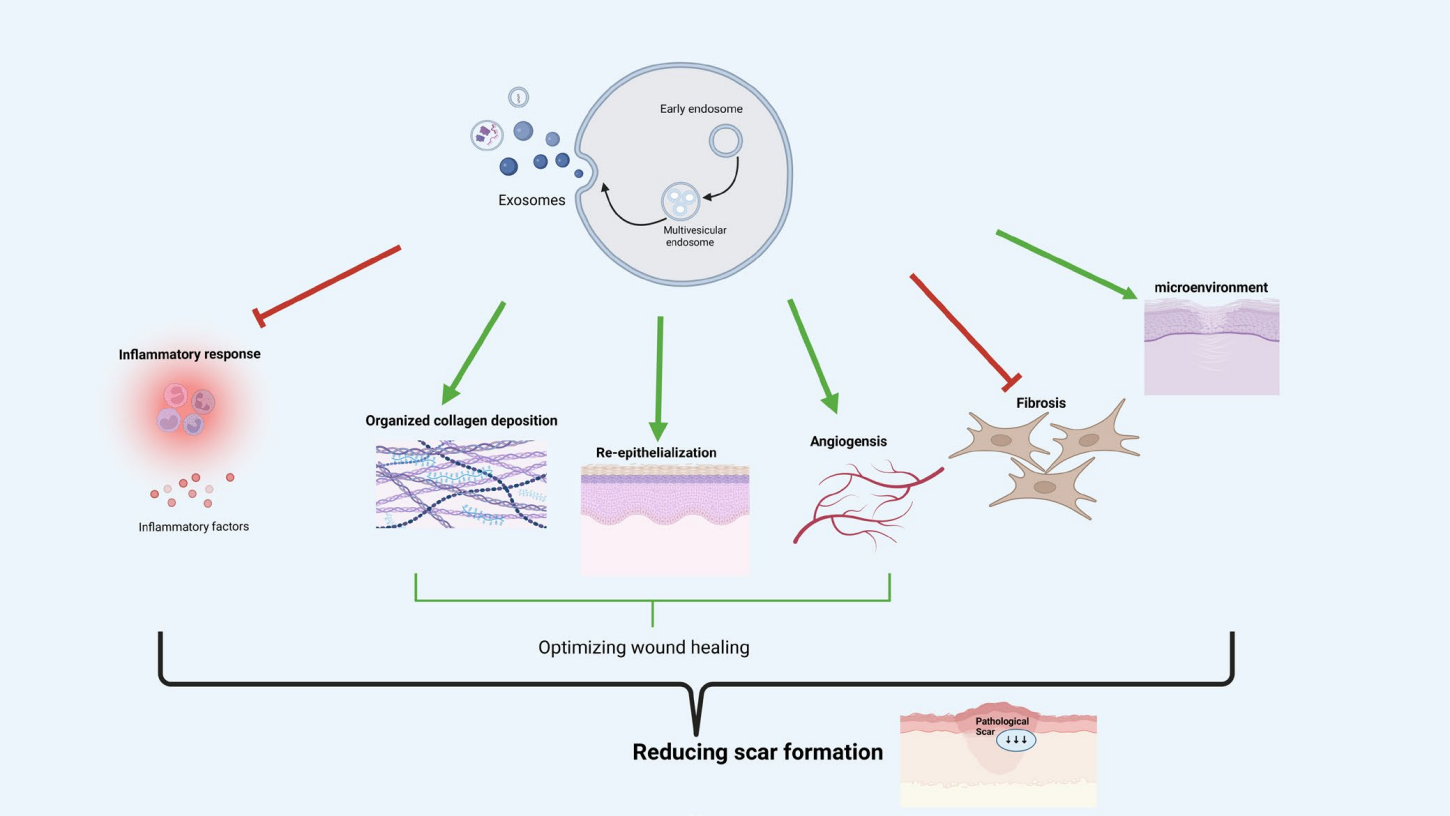
Exosomes regulate pathological scar formation by modulating inflammation, wound healing, fibrosis, and the scar microenvironment. This figure illustrates the multifaceted roles of exosomes in modulating inflammation (e.g., macrophage polarization, mast cell inhibition), optimizing wound healing (e.g., re‐epithelialization, angiogenesis, collagen deposition), inhibiting fibrosis (e.g., fibroblast differentiation, ECM homeostasis), and improving the local microenvironment (e.g., oxidative stress, neuro‐collagen interactions) in hypertrophic scars and keloids.

Persistent inflammation drives pathological scarring, including hypertrophic scars and keloids. Exosomes modulate inflammation by regulating immune cell activity and inflammatory factor release, influencing fibrosis progression. Figure 2 illustrates these regulatory mechanisms.

#### 3.1.1 Regulation of Macrophage Polarization and Autophagy

Macrophage polarization is critical for scar formation [43]. MSC‐Exos promote a shift from pro‐inflammatory M1 to reparative M2 macrophages, reducing inflammation and enhancing tissue repair [44, 45]. Specifically, ADSC‐Exos deliver long noncoding RNA (lncRNA) H19 to target the miR‐130b‐3p/PPARγ/STAT3 axis, driving M2 polarization and offering a therapeutic target for scar prevention [46].

Macrophage autophagy regulates immune responses and tissue repair via the autophagosome‐lysosome system [47]. Autophagy activation inhibits the nuclear factor‐κB (NF‐κB) signaling pathway by degrading the nucleotide‐binding oligomerization domain‐like receptor protein 3 (NLRP3) inflammasome, reducing p65 nuclear translocation and pro‐inflammatory cytokines, such as tumor necrosis factor‐α (TNF‐α) and interleukin (IL)‐1β [48]. Autophagy also activates PPAR‐γ and AKT/STAT3 pathways, promoting IL‐10 secretion and M2 polarization [49]. SHED‐Exos enhance autophagy flux via miR‐1246, targeting the AKT/ERK1/2 axis, and promote anti‐inflammatory and tissue repair effects [28].

#### 3.1.2 Inhibition of Mast Cell Activity

Mast cells have been identified as key players in inflammatory responses and tissue fibrosis [50, 51]. Clinical observations have further demonstrated a significant positive correlation between the number and activation level of mast cells and the severity of scarring [52]. Exosomes exhibit the ability to regulate mast cell activity through multiple mechanisms. For instance, tonsil‐derived MSC exosomes (tMSC‐Exos) inhibit the Toll‐like receptor (TLR) 7 signaling pathway, leading to a reduction in the release of inflammatory factors such as histamine and TNF‐α [53]. Additionally, recent studies have revealed that exosomes derived from human induced pluripotent stem cells (hiPSCs‐Exos) can suppress mast cell activation by modulating the hypoxia‐inducible factor (HIF)‐1 signaling pathway, offering a novel strategy for the prevention and treatment of pathological scarring [54].

#### 3.1.3 Regulation of T Cell Differentiation

T cell subsets regulate skin repair and fibrosis through complex immune networks [55], as summarized in Table 2. CD4+ T cell subsets have distinct roles: Th1 cells secrete interferon‐γ (IFN‐γ), inhibiting collagen deposition and promoting M1 macrophage polarization; Th2 cells promote fibroblast activation via IL‐4 and IL‐13; Th17 cells enhance inflammation and fibrosis through IL‐17A, with increased presence in keloid scars; and regulatory T cells (Tregs) maintain immune tolerance via IL‐10 and TGF‐β [56, 57]. CD8+ T cells support tissue homeostasis through Natural Killer Group 2D (NKG2D)‐mediated surveillance, but their tissue‐resident memory subset is dysfunctional in keloids [58]. γδ T cells and natural killer T (NKT) cells regulate repair via insulin‐like growth factor‐1 (IGF‐1) and CD1d‐restricted recognition, respectively [4].

**TABLE 2:** Impact of T cell subsets on scarring.

T cell subpopulation	Cytokine(s)	Effect on pathological scar formation
Th1	IFN‐γ	Inhibits
Th2	IL‐4, IL‐13	Promotes
Th‐20	IL‐17A	Promotes
Treg	IL‐10, TGF‐β	Inhibits
CD8+ T	IFN‐γ, TNF‐α	Promotes
γδ T	IGF‐1	Inhibits
NKT	IL‐4, IL‐13, IFN‐γ	Promotes/inhibits

*Note:* T cell subsets influence pathological scar formation through distinct cytokines.

UC‐MSC‐Exos and their small EVs restore T cell homeostasis by inhibiting pro‐inflammatory Th17 differentiation and promoting Treg expansion [59]. Their EVs deliver immune regulators, such as TNF‐α‐induced protein 6 and miRNA‐146a, suppressing T cell overactivation and reducing pro‐fibrotic factors (e.g., IL‐17A, TNF‐α), offering strategies for scar prevention [60].

#### 3.1.4 Regulation of Neutrophil Activity

Chronic neutrophil‐mediated inflammation in hypertrophic scars and keloids accelerates fibrosis [61]. Neutrophils release neutrophil extracellular traps (NETs), activating the TLR9/NF‐κB/IL‐6 pathway to promote myofibroblast differentiation and scar proliferation [62]. MSC‐Exos inhibit NET formation and pro‐inflammatory pathways by reducing CXCL 8 release and downregulating reactive oxygen species (ROS) and elastase expression, limiting neutrophil‐complement system activation [63, 64]. These effects suggest exosomes maintain scar microenvironment homeostasis.

#### 3.1.5 Regulation of Inflammatory Factor Release

Exosomes modulate inflammatory factor expression to alleviate chronic inflammation in scarring. UC‐MSC‐Exos reduce pro‐inflammatory cytokines (e.g., IL‐1β, TNF‐α) by inhibiting the TLR4/NF‐κB pathway while upregulating anti‐inflammatory IL‐10 [65]. Valproic acid‐loaded UC‐MSC‐Exos further suppress IL‐1β and TNF‐α expression [66]. These findings indicate MSC‐Exos balance pro‐ and anti‐inflammatory factors, offering a promising approach for scar management [65, 66].

### 3.2 Optimization of Wound Healing

#### 3.2.1 Regulation of Re‐Epithelialization

Accelerating re‐epithelialization is a critical strategy for mitigating pathological scar formation. Rapid epidermal regeneration serves as a primary defense by arresting wound exudation and reducing the risk of opportunistic infection [67]. Furthermore, the restoration of a functional skin barrier terminates persistent inflammatory stimuli, thereby blocking the aberrant signaling pathways that drive dermal fibroblast hyperactivation and excessive collagen deposition [67].

ADSC‐Exos promote re‐epithelialization via the lncRNA H19/miR‐19b/SOX9 pathway [68]. Under hypoxic conditions, ubiquitin‐specific protease 22 mediates HIF‐1α deubiquitination, upregulating lncRNA H19 in exosomes [69]. A meta‐analysis confirms ADSC‐Exos enhance re‐epithelialization and collagen arrangement [70]. Other MSC‐Exos, such as amniotic fluid‐derived stem cell exosomes (AFSC‐Exos), deliver miRNA‐146a‐5p to inhibit CXCR4 expression [71], while antler MSC‐derived exosomes (AnMSC‐Exos) activate the STAT3 pathway via miRNA‐21‐5p, promoting keratinocyte proliferation and migration [72]. Placental MSC‐derived exosomes (PMSC‐Exos) inhibit the Engrailed‐1 pathway to enhance keratinocyte activity [73], and amniotic membrane‐derived MSC exosomes (AMSC‐Exos) boost keratinocyte migration [74].

#### 3.2.2 Regulation of Angiogenesis

Sufficient and orderly angiogenesis provides essential oxygen and nutrients to support wound healing, promotes the formation of healthy granulation tissue, and helps regulate cellular metabolism and inflammatory responses within the local microenvironment [75]. Conversely, inadequate or disordered angiogenesis leads to hypoxia and chronic inflammation, which in turn activates myofibroblasts and promotes excessive ECM deposition, thereby aggravating scar hyperplasia [75].

Hypoxia‐induced ADSC‐Exos activate the PI3K/Akt pathway via vascular endothelial growth factor (VEGF) and miRNA‐126, promoting endothelial cell survival, proliferation, and migration [69, 76]. Umbilical cord blood‐derived exosomes (UCB‐Exos) enhance angiogenesis and matrix remodeling through miRNA‐21‐3p‐mediated PI3K/Akt activation [77]. Blue light pretreatment increases VEGF expression in UC‐MSC‐Exos, enhancing pro‐angiogenic activity [78]. MSC‐Exos inhibit endothelial cell ferroptosis via the miR‐17‐92 cluster, stabilizing neovascular networks [79]. Spatial transcriptomics shows MSC‐Exos and UCB‐Exos regulate the VEGF‐Notch pathway for functional vascular regeneration [80]. Keratinocyte‐derived exosomes treated with glycosaminoglycans upregulate VEGF and fibroblast growth factor expression, promoting vessel maturation [81].

#### 3.2.3 Promotion of Organized Collagen Deposition

Physiological wound healing involves sequential collagen deposition, transitioning from type III to type I collagen to form mature, regularly arranged fiber bundles. Exosomes regulate this process to prevent pathological scarring. UCB‐Exos deliver miRNA‐21‐3p to enhance collagen synthesis and matrix remodeling [77]. HiPSC‐Exos improve type I/III collagen synthesis and fiber arrangement [82]. ADSC‐Exos promote early collagen synthesis for wound closure while inhibiting α‐smooth muscle actin (α‐SMA) expression and excessive collagen deposition during fibrosis, balancing collagen metabolism [25, 83]. These mechanisms promote type I collagen‐dominated remodeling and regular fiber arrangement, reducing pathological scar formation.

### 3.3 Inhibition of Fibrosis

Hypertrophic scars and keloids result from excessive fibroblast activation and myofibroblast transformation, leading to abnormal ECM deposition. Exosomes inhibit fibrosis by targeting fibroblast behavior and ECM metabolism.

#### 3.3.1 Fibroblast‐Targeted Interventions

Exosomes block fibroblast‐to‐myofibroblast differentiation and reverse activated states. ADSC‐Exos inhibit the TGF‐β/Smad2 axis, reducing α‐SMA, LC3‐II, and Beclin‐1 levels [84]. Epidermal stem cell‐derived exosomes (EPSC‐Exos) downregulate TGF‐β1 via miR‐425‐5p and miR‐142‐3p, preventing myofibroblast transformation [85]. ADSC‐Exos deliver miR‐7846‐3p to target Neuropilin 2, suppressing the Hedgehog pathway and keloid fibroblast proliferation [86]. MSC‐Exos inhibit NF‐κB activation and α‐SMA expression via miR‐138‐5p targeting SIRT1 [87]. Bone marrow‐derived MSC exosomes (BMSC‐Exos) enhance p53 stability via lncRNA MEG3, inhibiting Minichromosome Maintenance Complex Component 5 transcription and fibroblast proliferation [88]. EPSC‐Exos deliver miR‐203a‐3p to inhibit PIK3CA, promoting myofibroblast dedifferentiation and offering a mechanism to reverse fibrosis [89].

#### 3.3.2 Regulation of ECM Homeostasis

Exosomes correct excessive ECM deposition by targeting synthesis and degradation pathways. ADSC‐Exos deliver miR‐29 and miR‐125b‐5p to inhibit collagen synthesis [25, 90]. MSC‐Exos induce fibroblast metabolic reprogramming, enhancing glycolysis and suppressing oxidative phosphorylation to reduce ECM deposition [91]. ADSC‐Exos balance matrix metalloproteinases (MMPs) and tissue inhibitors of metalloproteinases (TIMPs), enhancing ECM degradation and promoting scar‐free repair [92].

### 3.4 Optimization of Local Microenvironment

#### 3.4.1 Alleviation of Oxidative Stress

Oxidative stress, characterized by excessive ROS, drives fibroblast proliferation, myofibroblast transformation, and collagen deposition in scarring [93]. ADSC‐Exos mitigate oxidative stress by reducing miR‐486‐3p expression in H₂O₂‐treated models, inhibiting SIRT6 and Smad signaling to limit ROS bursts and endothelial‐to‐mesenchymal transition [93]. In UVB‐induced photoaging models, ADSC‐Exos reduce ROS and pro‐inflammatory cytokines (e.g., IL‐6, TNF‐α), inhibiting fibrosis [94]. MSC‐Exos suppress neutrophil‐complement system activation, downregulating ROS and supporting microenvironment homeostasis [64, 95].

#### 3.4.2 Neuro‐Collagen Interactions

Wound‐induced nerve damage disrupts sensory signaling, with inflammation, ischemia, and fibrosis reducing neurotrophic factor secretion, delaying healing [96]. UC‐MSC‐Exos promote neural repair by: (1) enhancing fibroblast migration; (2) upregulating neurotrophic factor genes (e.g., TAC4, TAC2, CALCB, VIPR1, TACR1); (3) promoting axon growth, Schwann cell proliferation, and nerve fiber myelination; and (4) improving wound blood supply via fibroblast and endothelial cell regulation [27].

## 4 Molecular Mechanisms of Exosomes' Regulation of Scarring

**FIGURE 3:**
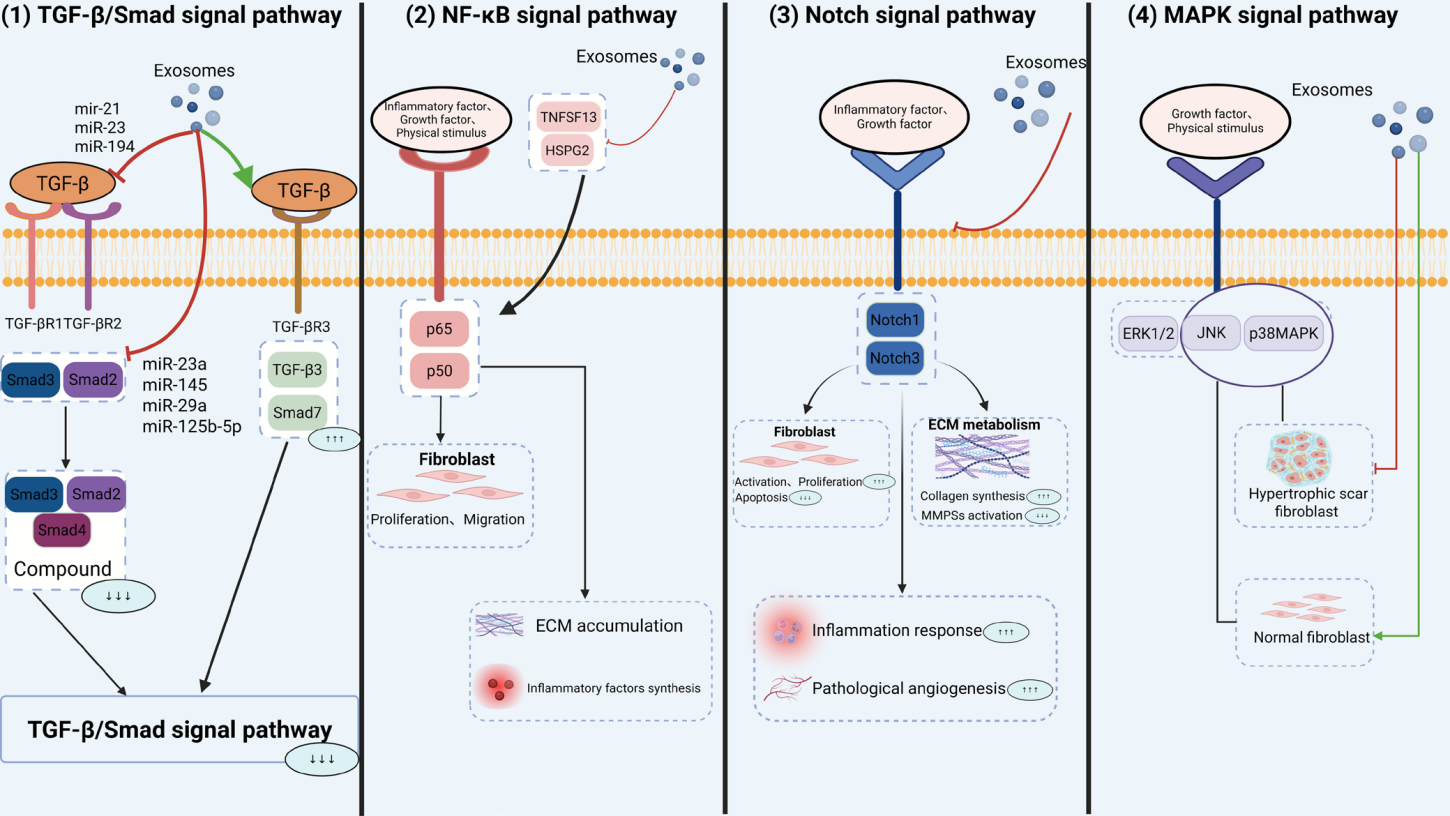
Exosomes regulate scarring via TGF‐β/Smad (e.g., miR‐125b‐5p targets Smad2), NF‐κB (e.g., TNFSF13/HSPG2 inhibition), Notch (e.g., Notch‐1 downregulation), and MAPK (e.g., p‐JNK/p38 MAPK regulation). This figure depicts the molecular pathways modulated by exosomes in scar formation, including TGF‐β/Smad (e.g., miRNA‐125b‐5p targeting Smad2), NF‐κB (e.g., TNFSF13/HSPG2 inhibition), Notch (e.g., Notch‐1 downregulation), and MAPK (e.g., bidirectional p‐JNK/p38 MAPK regulation). Key interactions, such as receptor binding and gene transcription, are shown to highlight exosomes' role in reducing fibrosis and promoting wound healing in pathological scars.

Exosomes regulate scar formation through key signaling pathways, including TGF‐β/Smad, NF‐κB, Notch, and MAPK, as illustrated in Figure 3.

### 4.1 TGF‐β/Smad Signaling Pathway

The TGF‐β signaling pathway is a primary regulator of the balance between wound healing and scar formation. Among its isoforms, TGF‐β1 and TGF‐β2 are overexpressed in keloid fibroblasts and function as potent drivers of fibrosis [97, 98]. In contrast, TGF‐β3 is typically associated with reduced collagen deposition and improved scar architecture, although its expression remains low in most wounds and may paradoxically exacerbate ECM deposition under certain pathological conditions [97, 98]. This signaling cascade is mediated by Smad proteins, which form transcriptional complexes upon binding to Type I/II receptors to regulate collagen synthesis [99].

Exosomes from various cellular sources precisely intercept this pathway to mitigate scarring: (1) ADSC‐Exos: Adipose‐derived stem cell exosomes rebalance ECM synthesis by downregulating TGF‐β2 and phospho‐Smad3 while elevating TGF‐β3. Furthermore, they deliver miR‐125b‐5p to directly target the 3′UTR of Smad2, silencing its expression and halting downstream fibrotic transcription [25, 100]. (2) AFSC‐Exos: Exosomes from human amniotic fluid stem cells carry the let‐7‐5p family, which inhibits TGF‐β receptors I and II, effectively attenuating myofibroblast differentiation [101]. (3) UCB‐Exos: Umbilical cord blood MSC exosomes leverage miR‐21‐5p and miR‐125b‐5p to block TGF‐β receptors, further interrupting the Smad‐dependent signaling cascade [102].

### 4.2 NF‐κB Signaling Pathway

The NF‐κB pathway serves as a central regulator of inflammation and fibrosis, playing a pivotal role in the pathogenesis of hypertrophic scars and keloids [103]. In hypertrophic scarring, the cytokine TNFSF13 is upregulated and binds to heparan sulfate proteoglycan 2 (HSPG2). This interaction triggers the phosphorylation and nuclear translocation of the p65 subunit, which subsequently drives the transcription of pro‐fibrotic and inflammatory genes [103].

MSC‐Exos can effectively intercept this fibrotic signaling axis. Specifically, MSC‐Exos downregulate the expression of both TNFSF13 and HSPG2 in hypertrophic scar fibroblasts, leading to a significant reduction in p65 phosphorylation and its associated nuclear activity [104]. This inhibition results in the attenuation of fibroblast proliferation and migration, as well as a decrease in key fibrotic markers such as α‐SMA and COL1A1. Consequently, this highlights a targeted molecular mechanism through which exosomes mitigate scar‐associated inflammation and pathological ECM deposition [104].

### 4.3 Notch Signaling Pathway

The Notch signaling pathway, particularly the Notch‐1 receptor, is a critical driver of fibroblast proliferation and pathological angiogenesis during scar formation [105]. In keloids, aberrant activation of this pathway promotes excessive ECM deposition and tissue stiffening.

Exosomes provide a targeted approach to modulating this signaling network. Specifically, ADSC‐Exos have been shown to effectively downregulate Notch‐1 expression in keloid fibroblasts [100]. This inhibition creates a synergistic effect by reducing the levels of TGF‐β2 and phosphorylated Smad3, which in turn decreases the synthesis of major fibrotic components, including Collagen I and III, Fibronectin, and α‐SMA [100]. By simultaneously disrupting the Notch‐1 axis and the interconnected TGF‐β2/Smad3 pathway, ADSC‐Exos effectively dismantle a key fibrogenic network, demonstrating their therapeutic potential to mitigate pathological ECM accumulation [100].

### 4.4 MAPK Signaling Pathway

The Mitogen‐Activated Protein Kinase (MAPK) pathway—comprising the ERK, JNK, and p38 sub‐pathways—is a fundamental regulator of cell proliferation, differentiation, and fibrotic responses [106]. Dysregulation of this axis is a primary driver of fibroblast hyperactivation and excessive collagen synthesis in pathological scarring [107].

A particularly compelling therapeutic mechanism has been observed with bacterial extracellular vesicles derived from *Lactobacillus druckerii* (LDEVs), which exhibit a bidirectional modulatory effect on MAPK signaling depending on the cellular context. In Hypertrophic Scar Fibroblasts: LDEVs downregulate the levels of phosphorylated JNK (p‐JNK) and p‐p38. This inhibition effectively suppresses cell proliferation and the expression of Collagen I and III [29]. In Normal Fibroblasts: Conversely, LDEVs upregulate these same phosphorylated proteins, thereby supporting physiological wound healing and tissue maintenance [29].

This cell‐type‐specific regulation is a significant finding; it suggests that exosome‐based therapies can be engineered to selectively silence fibrotic signaling in pathological cells while simultaneously preserving or even enhancing reparative functions in healthy tissue.

## 5 Discussion

Exosomes, key paracrine mediators in the dermal microenvironment, play a critical role in the pathophysiology of hypertrophic scars and keloids, underscoring their potential for research and therapy. These EVs regulate pathological scar progression through multiple mechanisms, including modulating inflammation, promoting wound healing, inhibiting fibrosis, and optimizing the local microenvironment. Emerging evidence highlights their dual potential as biomarkers for scar diagnosis and as drug delivery carriers. Their low immunogenicity and high biocompatibility make them promising candidates for cell‐free and gene therapies.

Engineered exosomes represent a sophisticated evolution in scar therapy, offering a more targeted and controllable strategy than their native counterparts [108]. Through surface modification, cargo loading, or membrane fusion, these vesicles can be customized to enhance their accumulation within scar tissue and improve drug delivery efficiency [108]. This precision allows for the exact regulation of specific pathways, such as the TGF‐β/Smad axis, while minimizing off‐target effects.

By addressing the inherent limitations of natural exosomes—specifically regarding batch‐to‐batch homogeneity and therapeutic reproducibility—engineered versions provide a standardized pharmacological profile [109]. This shift from discovery to bioengineering is a critical step in overcoming the hurdles of clinical translation, paving the way for more reliable and potent anti‐fibrotic treatments [109].

Despite significant progress in exosome research, clinical translation faces challenges, including inefficient isolation and purification, heterogeneity among exosome subtypes, and variable therapeutic reproducibility. To overcome these hurdles, future studies should focus on optimizing large‐scale exosome production and developing stable, scalable therapeutic strategies. Additionally, exploring exosome heterogeneity in skin diseases and their molecular cargo could identify new therapeutic targets and enhance treatment predictability.

In conclusion, exosome research has advanced our understanding of hypertrophic scar and keloid pathogenesis, providing novel insights for clinical management. Future efforts should prioritize three areas: elucidating exosome mechanisms in scar formation, enhancing their therapeutic efficacy, and achieving clinical translation. These advancements could enable precise, effective treatments for pathological scars, though further validation in clinical trials is needed.

## Author Contributions

M.W. and J.Z. contributed equally as first authors, leading conceptualization, literature review, and drafting. N.X., Y.M., and L.Y. collected literature and revised the manuscript. H.L. and Q.G., as corresponding authors, oversaw the project and ensured scientific rigor. Q.G. handled submission.

## Funding

This study was funded by the Natural Science Foundation Project of Nanjing University of Chinese Medicine (grant number: XZR2024005).

## Ethics Statement

The authors confirm that the ethical policies of the journal, as noted on the journal's author guidelines page, have been adhered to. No ethical approval was required as this is a review article with no original research data.

## Consent

The authors have nothing to report.

## Conflicts of Interest

The authors declare no conflicts of interest.

## Data Availability

Not available—no new data generated, or the article describes entirely theoretical research.
